# Environmental Monitoring of Ancient Buildings Based on a Wireless Sensor Network

**DOI:** 10.3390/s18124234

**Published:** 2018-12-02

**Authors:** Qijing Lin, Fuzheng Zhang, Weile Jiang, Hao Wu

**Affiliations:** 1 Collaborative Innovation Center of High-End Manufacturing Equipment, Xi’an Jiaotong University, Xi’an 710054, China; qjlin2015@xjtu.edu.cn; 2 State Key Laboratory for Manufacturing Systems Engineering, Xi’an Jiaotong University, Xi’an 710049, China; xjzfz123@stu.xjtu.edu.cn (F.Z.); wuhao713@stu.xjtu.edu.cn (H.W.); 3 State Key Laboratory of Mechanical System and Vibration, Shanghai Jiao Tong University, Shanghai 200240, China; 4 Institute of Heritage Sites & Historical Architecture Conservation, Xi’an Jiaotong University, Xi’an 710049, China; 5 School of Architecture, Xi’an University of Architecture and Technology, Xi’an 710055, China

**Keywords:** ancient buildings, ZigBee, environment monitoring, package, monitoring platform, over-limit warning

## Abstract

Environmental monitoring plays an important role in the preventive protection of ancient buildings, although it is still in the prototype stage. In order to provide data support for the protection of ancient buildings, an environmental monitoring system with multi-sensor and multi-node for the interior and exterior of ancient buildings is designed and realized, based on ZigBee, TCP/IP, and intranet penetration technology. The new type of indoor node package box and outdoor package device are designed to meet the needs of different types of sensors. The monitoring platform is developed on the strength of the LabView so as to obtain real-time display, storage, and over-limit warning functions for local and remote monitoring data. It also proves that the monitoring system is stable and reliable by monitoring the actual ancient building with a brick (stone) structure.

## 1. Introduction

An ancient building refers to a building left by the ancients with historical value, and is the crystallization of the wisdom of the ancient working people. It is the precious historical materials for studying the political economy, cultural art, and religious belief of ancient society, and the precious historical and cultural heritage that is non-renewable. Most importantly, ancient buildings are an important symbol of national civilization [1,2]. However, with the erosion of thousands of years and the deterioration of the natural environment, a large number of ancient buildings have been destroyed. In particular, some ancient buildings in remote areas that lack effective and timely human protection are not optimistic, and urgently need the effective protection. At present, with the full speed development of social science and technology, information technology has been popularized and applied to all aspects of cultural heritage, such as the preservation of cultural relics, protection and restoration of ancient buildings, and so on [3,4]. In the field of cultural heritage, the most striking feature of information technology is its applicability. Integrating information technology and protection of cultural heritage is an important research topic that deserves careful study and exploration.

When formulating the protection and restoration of the internal environment of ancient buildings, we must consider the impact of the external environment and the human activities on it. With the gradual increase of the degree of governmental supervision and the continuous improvement of people’s cultural quality, the phenomenon of artificially destroying ancient buildings is happening less and less. However, the influence of environmental factors on ancient buildings is becoming more and more important. This effect is not achieved overnight, but is accumulated over time. The protection of cultural relics is no longer only concerned with the individual, but begins to pay attention to the whole, that is, the impact of the environment on cultural relics [5]. The environmental parameters of ancient buildings include temperature, relative humidity, carbon dioxide, pH, wind speed, and so on. For example, change of temperature and relative humidity have a major impact on ancient buildings. For ancient buildings with wooden structures, these will be damaged and deformed because of extreme heat or cold. Excessive relative humidity will cause decay, moth, mildew, and other phenomena in wooden structures, which will greatly reduce the seismic performance of the unique wood frame of ancient buildings. For ancient buildings with brick (stone) structures, the influence of ambient temperature and relative humidity are mainly the cause of corrosion. In addition, the high temperature and relative humidity will also lead to the corrosion of metal parts, reduction of lightning protection capability, fading of wood carvings, and so on. Acid rain has a strong corrosive effect on brick (stone) buildings with carbonates, which accelerates the aging process of ancient buildings. Many ancient buildings and stone carvings in the world have been damaged by acid rain, such as the Leshan Giant Buddha in China and the Parliament Building in Canada. Dust is a very fine particle that easily invades the gaps of murals and statues, thus polluting the artistic visual effects of carvings, murals, and so on. In addition, the deposition of dust will destroy the adhesion of the murals, and cause large-scale detachment and fading [6]. Sulfur dioxide has a destructive effect on cultural relics with limestone. The damage caused by tiny vibrations is not obvious [7], but long-term vibrations will cause a large number of cracks in the surface layer of the rock mass, such as in the Longmen Grottoes and Yungang Grottoes in China, resulting in large-scale shedding. Therefore, the influence of environmental factors on ancient buildings is very large. It is of great significance for the protection of ancient buildings to obtain the environmental information.

Wireless sensor network (WSN) is a hot research field and is highly valued by various countries. Furthermore, it has the advantage of high information integration and involves multiple disciplines [8,9,10,11,12]. The network protocols applied to WSN mainly include WiFi, Bluetooth, UWB, NFC, GPRS, ZigBee, and so on. ZigBee is widely used in smart agriculture [13,14,15], chemical detection [16,17], medical [18], and other fields [19], and has a low complexity, self-organizing network, low cost, low power consumption, and high security. Wu et al. [20] introduced a hospital intelligent smoke detection system using ZigBee transmission technology. Compared with the traditional smoke detection system, it has a higher accuracy and controllability, and adds visual capabilities for monitoring data. However, the monitoring parameter is single and the system scale is small. Based on ZigBee and WSN, Leccese et al. [21] described a new intelligent street lighting system that integrates new technologies available on the market, in order to offer higher efficiency and considerable savings. Chen et al. [22] designed an excellent wireless monitoring system of the mountain agriculture based on the ZigBee wireless transmission network. The system realized the real-time collection of environmental parameters such as the temperature, relative humidity, and illumination of unattended agricultural bases. Pérez-Garrido et al. [23] proposed a remote monitoring system for toxic gases in the shipbuilding industry. The system works normally and is easy to install, but the monitoring software has not been not designed. Gong et al. [24] designed an environment monitoring system for smart homes based on ZigBee. The hardware design is carried out and the monitoring system is in real-time, but the monitoring parameters are too small. Furthermore, the monitoring system has no data storage and early warning functions, and there is no package design. In order to monitor and control the pH value of water in thermal power plants, Huang et al. [25] designed a smart pH analyzer based on ZigBee. The CC2430 is used to establish the wireless network. The system has a high accuracy and reliability, but the monitoring parameter is single. Unfortunately, at present, swireless sensor network are rarely used in the field of ancient building environmental monitoring, although there are also many researchers who have done a lot of work regarding this [26,27,28,29,30,31]. Leccese et al. [32] designed a new acquisition system for the remote control of murals and tested it in the field. The environmental parameters, such as temperature and atmospheric pressure, obtained from the local monitoring, are transferred remotely, allowing for an easier management by experts in the field of conservation of cultural heritage; Mesas-Carrascosa et al. [33] designed a system to monitor the microclimate variables in heritage buildings by using open source hardware. The behavior of the interior temperature and relative humidity in relation to this was also assessed. Zhang et al. [34] proposed a micro-environment control system composed of automatic partition doors and WSN. The temperature, relative humidity, and carbon dioxide concentration in the Mogao Grottoes in Dunhuang were measured. However, the monitoring parameters are relatively less, and the package structure and the user monitoring platform are also relatively simple. Aparicio et al. [35] provided a performance evaluation of the tree and mesh routing topologies of WSN in San Juan Bautista Church in Talamanca de Jarama (Madrid, Spain). Moreover, low energy adaptive clustering hierarchy (LEACH) can apply Dijkstra’s shortest path algorithm to select the optimum path [36]. Based on GPRS technology, Agbota et al. [37] described an environmental monitoring prototype designed to assess the impact of the environment on cultural heritage. This prototype consists of an array of piezoelectric quartz crystals and on a temperature and relative humidity sensor. However, the limitation of the monitoring system is obvious, because this system can only monitor specific materials. Based on the JenNet protocol of WSN, Zhao et al. [38] designed an over-limit warning system for electrical fire to be used in ancient buildings. This system analyzes and solves the wiring problem, but there are also problems with its single monitoring parameter and lack of monitoring software. As the “Internet+” age is coming, the information technologies, such as wireless sensor networks, big data, and cloud computing, will play a larger role in the field of protection and restoration of ancient buildings.

In order to provide data support for the protection of ancient buildings, an environmental monitoring system with multi-sensor and multi-node based on ZigBee, TCP/IP, and intranet penetration technology, is designed and implemented, which can be used for the internal and external, short-range, and remote monitoring of ancient buildings. The designed monitoring system realizes the real-time monitoring of environmental parameters in ancient buildings, such as temperature, relative humidity, fine particles (PM2.5), carbon dioxide, carbon monoxide, sulfur dioxide, vibration, liquefied gas, wind speed, wind direction, and the pH of rainwater. Based on the LabView, the user monitoring platform is designed and developed to realize the real-time display, storage, and over-limit warning functions for local and remote monitoring data. A new type of indoor node package box is designed on the strength of the transparent acrylic sheets, in order to protect against dust and water. A dust-proof and breathable outdoor package device is also provided. Moreover, the monitored environmental parameters are not only important for ancient buildings, but are also significant for tourists and staff in ancient buildings. By monitoring the environmental parameters of ancient buildings, this system provides the necessary data support for the conservation of ancient buildings, and ensures that people are in a good environment.

## 2. Materials and Methods

### 2.1. Hardware

#### 2.1.1. ZigBee Nodes

Anliande EB2530 boards are used for data acquisition and its subsequent submission to the computer, because of their ease of implementing a communication protocol. The core component of the node module is the CC2530 chip produced by TI, which integrates a microcontroller, an ADC, and a wireless communication module [39]. The single-chip microcomputer is an 8-bit 8051 microcontroller core of the enhanced industry standard. The core of the wireless communication module complies with the IEEE 802.115.4/ZigBee protocol, and supports CRC hardware verification. The ZigBee nodes can be divided into terminal devices, routers, and coordinators. Each ZigBee network only needs one coordinator. The terminal device, router, and coordinator are not different in structure, but they show different functions, because their codes are different.

#### 2.1.2. Sensors

This monitoring system has a multi-node and multi-sensor, which is designed to monitor environmental parameters, such as the temperature, relative humidity, PM2.5, carbon dioxide, carbon monoxide, sulfur dioxide, vibration, liquefied gas, wind speed, wind direction, and pH of rainwater in ancient buildings. The types of sensors and technical parameters used in this system are shown in Table 1. The terminal devices are divided into three categories based on the types of sensors connected to them. The connection between the signal output of the sensor and the signal input of the terminal device is shown in Table 2.

#### 2.1.3. Power Supply System

The power supply modules are mainly divided into indoor and outdoor parts. The indoor terminal devices and routers are usually powered by a constant voltage source. However, they can also use the AA battery to supply power in special cases such as during power failure. The battery pack has an output voltage of 5 V, which matches the input voltage of the terminal devices or the routers. The coordinator is directly connected to the PC via a USB cable, so it is powered by a constant voltage source.

Because of the relatively complex conditions of the outdoor environment, the outdoor nodes use a small solar power system to supply power. The controller is connected to the solar photovoltaic panel and the storage battery. When the solar photovoltaic panel is exposed to the sun, the generated electricity can be transferred to the storage battery through the controller, or it can be output from the controller to the load. The voltage output from the controller is 5 V, which is sufficient for the terminal device and the router to work normally. However, in the case of an insufficient solar power supply due to bad weather, the battery pack can also be used for temporary power supply.

### 2.2. System Design

#### 2.2.1. Overall Design of the System

**Figure 1:**
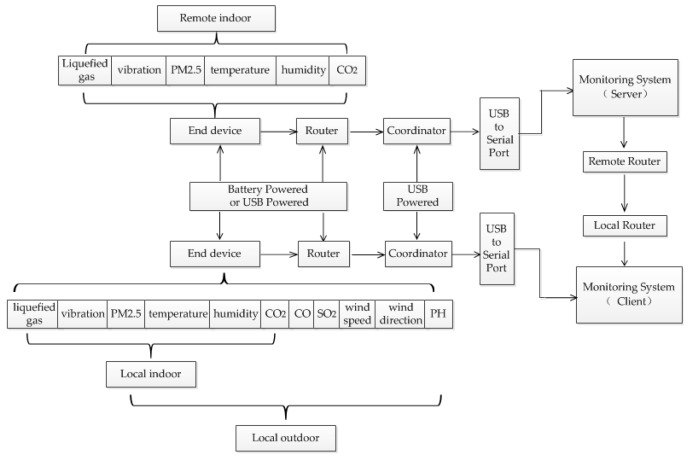
The layout of overall system.

**Figure 2:**
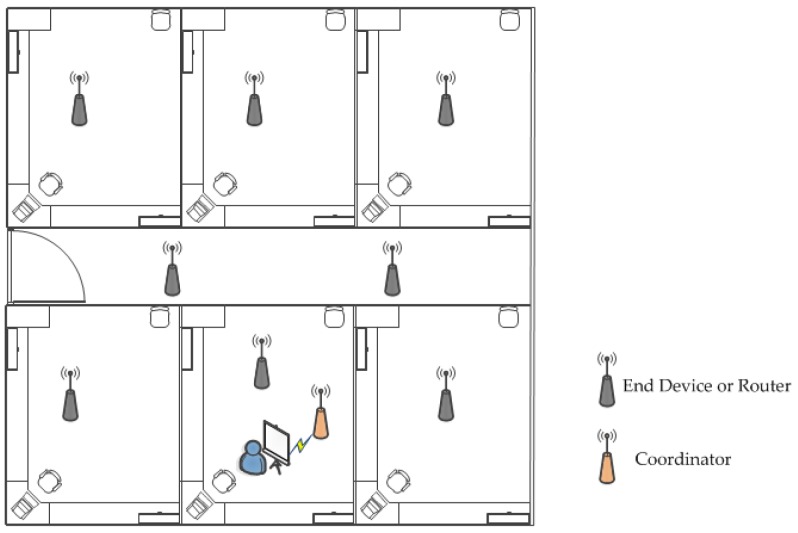
The plan of indoor monitoring.

The system is divided into three parts—indoor monitoring, outdoor monitoring, and remote monitoring. Each indoor terminal device includes a temperature and relative humidity sensor, PM2.5 sensor, liquefied gas sensor, vibration sensor, and carbon dioxide sensor. The outdoor terminal device includes a temperature and relative humidity sensor, PM2.5 sensor, carbon monoxide sensor, carbon dioxide sensor, sulfur dioxide sensor, wind speed sensor, wind direction sensor, and pH sensor. As shown in Figure 1, the data collected by the terminal device is finally transmitted to the coordinator through a series of routers. The coordinator is connected to the PC by a USB cable, and the remote data (server) can also be transferred to the local monitoring platform (client) via the local area network (LAN). The plan of indoor monitoring is shown in Figure 2.

#### 2.2.2. Network Topology

The communication method of the ZigBee adopted by this system is the broadcast. After collecting the environmental data, the terminal device broadcasts the data to all of the ZigBee nodes in the network. The router in the network automatically selects and accepts the information through the strength of the signal, and then propagates them to other routers. The code to set the broadcast is as follows:

SampleApp_Periodic_DstAddr.addrMode = (afAddrMode_t)AddrBroadcast; // broadcast.
SampleApp_Periodic_DstAddr.endPoint = SAMPLEAPP_ENDPOINT; //Specify endpoint number.
SampleApp_Periodic_DstAddr.addr.shortAddr = 0xFFFF; // Specify the destination network address as the broadcast address.

**Figure 3:**
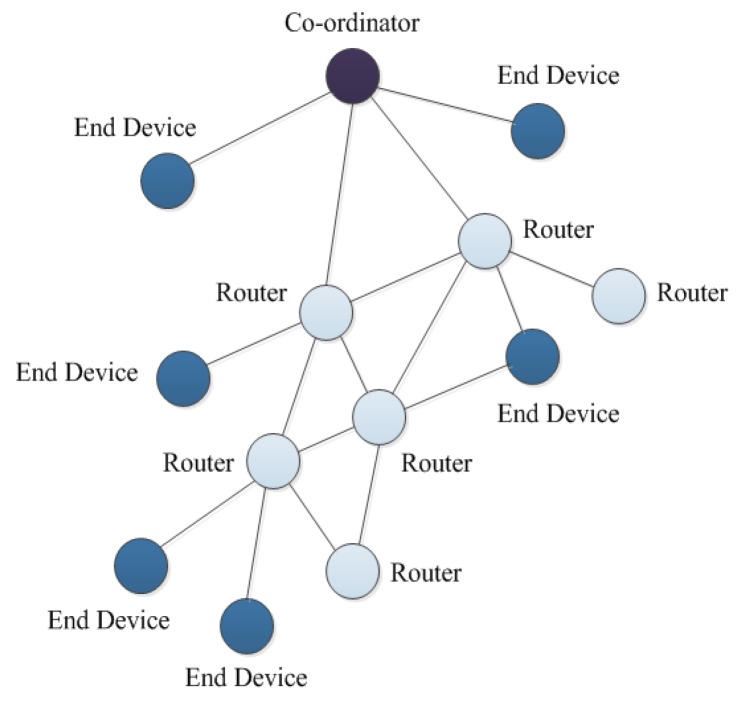
The schematic of mesh topology.

The topology used in this system is a mesh structure. Compared with the star structure and tree structure, the mesh structure is self-healing and has a high extension and reliability [40,41]. As shown in the mesh topology of Figure 3, there may be multiple routers between one router and another router. These redundant routers greatly increase the stability of the network, while providing more routings to the terminal devices. The mesh structure has a strong ability to adapt to the environment. The multiple routers in the network have the ability to re-route, which ensures that one of the damaged routers will not affect the operation of the entire network.

#### 2.2.3. Remote Communication

**Figure 4:**
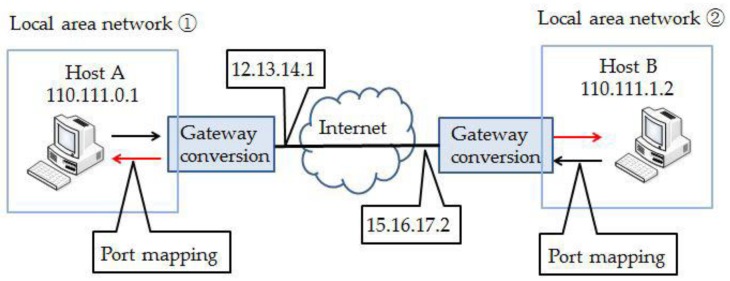
The schematic of intranet penetration technology.

The method of remote communication used by this system is intranet penetration technology. To put it simply, the technology of communication between hosts in different local area networks (LANs) via the Internet is called intranet penetration. As shown in Figure 4, when host A communicates with host B, belonging to another LAN, host A cannot directly accesses host B’s private IP 110.111.1.2. In order to achieve communication between two hosts in different LANs, we need to realize port mapping between public network IP 15.16.17.2 in LAN ② and the private IP 110.111.1.2 of host B. In this way, host A can automatically forward the IP datagram to the host B by sending the IP datagram with the destination address of 15.16.17.2.

### 2.3. Packaging Structure

#### 2.3.1. Indoor Node Package Box

The indoor node module mainly includes a temperature and relative humidity sensor, PM2.5 sensor, carbon dioxide sensor, liquefied gas sensor, vibration sensor, EB2530 board, and so on. Based on the layouts of the devices and better functions, the indoor node package box needs to comply with the following design requirements:

- Miniaturization. This is good for moving and installing, saving indoor space, and avoiding an impact on the other items in the room.
- Regionalization. Because the node contains many devices, placing the modules separately is beneficial in order to avoid the problem of entanglement between the wires.
- Ventilation. As each sensor needs to be in contact with outside air in order to achieve the measurement function, the package design must ensure the circulation of air.
- Waterproofness. This is mainly for the waterproof protection of the terminal node.
- Aesthetics. Aesthetics is one of the general design requirements.

#### 2.3.2. Outdoor Package Device

The outdoor environment is more complicated than the indoor environment. Moreover, the outdoor node module also includes many larger devices such as a wind speed sensor, wind direction sensor, pH sensor, and solar self-powered system. Also, because the nodes are placed outdoors, they are inevitably affected by factors such as the weather, people, vehicle flow, and so on. Therefore, in addition to meeting the requirements of miniaturization, regionalization, ventilation, waterproofness, and aesthetics, the package design of the outdoor nodes must comply with the following requirements:

- The package device needs to realize the functions of collecting and exporting rainwater in order to achieve the measurement of the pH of the rainwater.
- Non-interfering installation for large sensors. Large sensors such as wind speed sensors and wind direction sensors need to be properly installed in a limited space.
- The installation of a solar self-powered system. As the outdoor nodes may be in an unattended environment for a long time, it is necessary to ensure the continuous supply of electricity in order to enable them to work uninterruptedly.

### 2.4. Software Design

The design and development of a software system based on LabView is essentially the design integration of various functional modules. The software system includes the following five functional modules that can complete various tasks: “data acquisition”, “data display”, “data storage”, “system help”, and “remote communication”.

**Figure 5:**
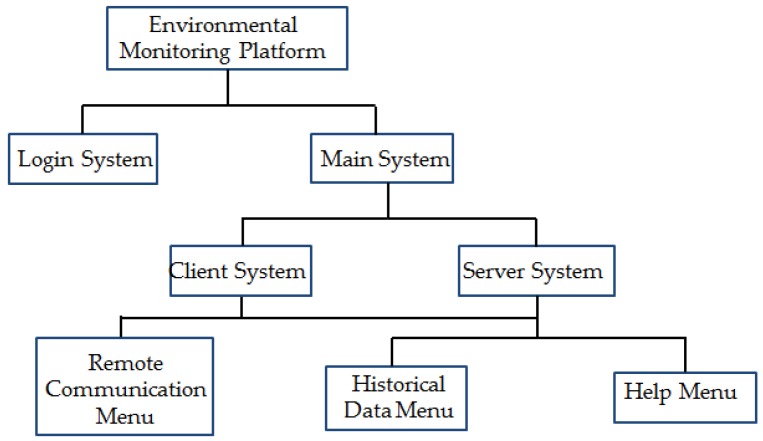
The structural relationship of software system.

As shown in Figure 5, the software system is mainly composed of the login system and the main system, and the main system is further divided into the client system and the server system. The login system mainly implements the functions, such as login management and the modification of the account and password. The collected data can be filtered by the server system in order to eliminate the error data. The server system can also realize the real-time display, storage, and over-limit warning of data, as well as transmit the collected data to the client system. The server system contains the historical data menu and the help menu. Users can view the data stored in the background through the historical data menu, which is convenient for users to summarize and analyze. The help menu can provide the operating guides of the system for users, which helps users to use the software. In addition to the functions of the server system, the client system also has the remote communication menu that can receive the data from the server system.

## 3. Results

### 3.1. Packaging Structure

#### 3.1.1. Indoor Node Package Box

**Figure 6:**
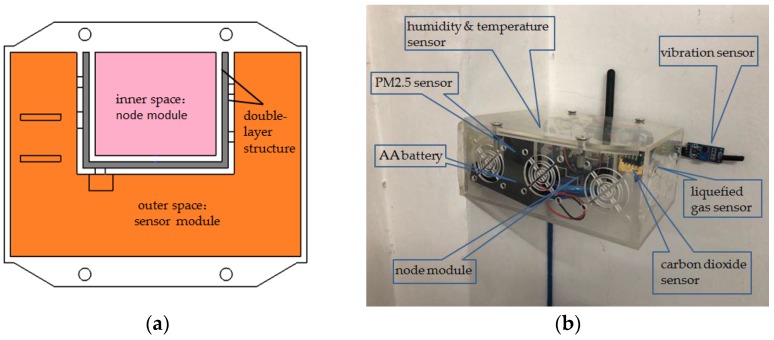
The design of indoor node package box: (**a**) schematic of the structure; (**b**) picture of the real product.

As shown in Figure 6a, the material used in the indoor node package box of the system is the transparent acrylic plate. The packaging structure is spliced by the organic adhesive (acrylic glue) and has the advantage of convenient design, simple assembly, low cost, and so on. In addition, the transparent acrylic plate allows the user to directly observe the operation of each sensor from the outside. More importantly, the layered structure design can isolate the node module from the sensor module, which can not only protect the node module from dust and water, but also facilitate the replacement in the case of damage to the sensor module. The installation position of each sensor is shown in Figure 6b. The terminal device is placed at the corner of the wall, which can minimize the impact of the package structure on the monitoring environment.

#### 3.1.2. Outdoor Package Device

**Figure 7:**
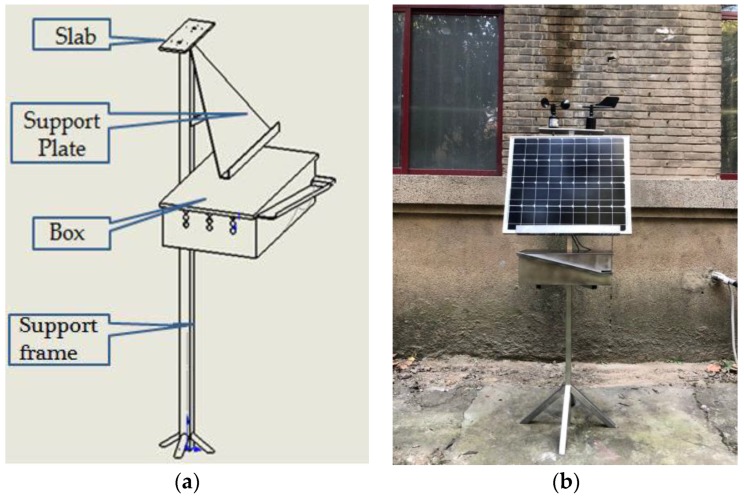
The design of outdoor package device: (**a**) picture of the structure; (**b**) picture of the real product.

Based on the previous design requirements, the outdoor package device is made of a steel plate and is firm and reliable. As shown in Figure 7, it can be roughly divided into the following four parts:

- Slab: place the wind speed sensor and the wind direction sensor.
- Support plate: place the solar photovoltaic panels.
- Box: lace the node modules and achieve the collection and automatic drainage of the rainwater.
- Support frame: mainly support the entire device.

**Figure 8:**
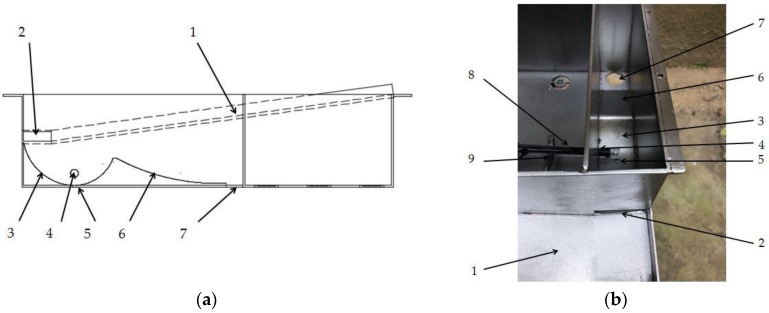
The device of rainwater collection and discharge: (**a**) picture of the structure; (**b**) picture of real product: (1) groove of rainwater collection; (2) drain outlet; (3) rainwater storage tank; (4) fixed orifice; (5) drain hole; (6) drainage trough; (7) drain hole; (8) pH sensor; (9) fixed collar.

The new device that we designed for rainwater collection and discharge is shown in Figure 8. The rainwater flows into a storage tank through the groove of rainwater collection and drain outlet, and is then discharged through the drainage trough and drain hole into the outside of the device. This structure is simple, but very ingenious. For the rainwater storage tank, it is a structure with one high side and one low side, which can ensure the measurement of the pH sensor and facilitate the discharge of rainwater. In the end, the rainwater accumulated in the storage tank is finally discharged through the drain hole.

### 3.2. Monitoring System

#### 3.2.1. Monitoring Platform

**Figure 9:**
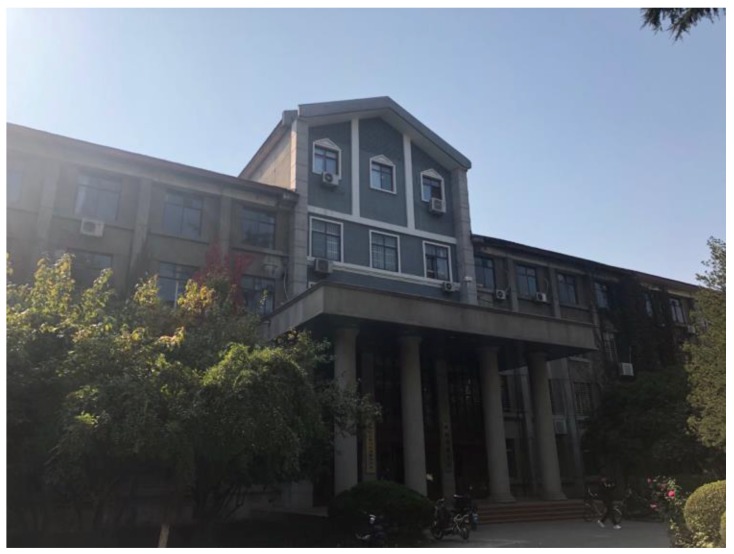
The cultural relics protection unit of Shaanxi Province.

**Figure 10:**
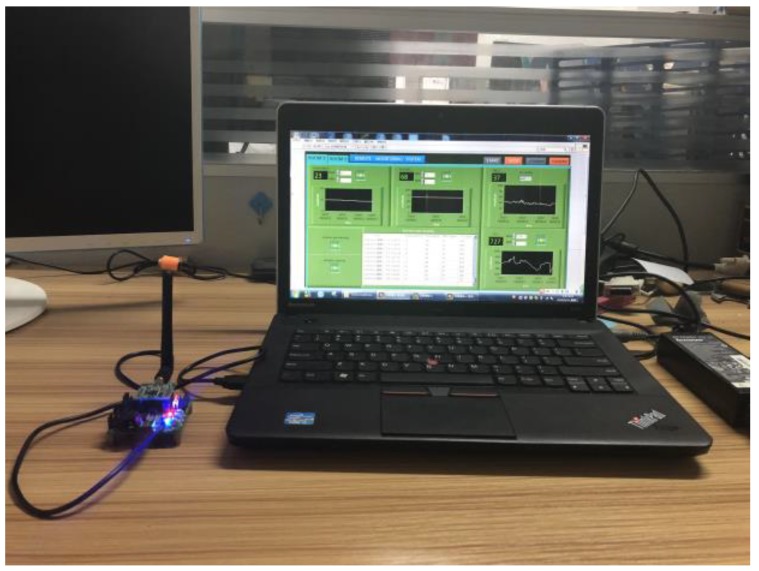
The remote monitoring platform.

**Figure 11:**
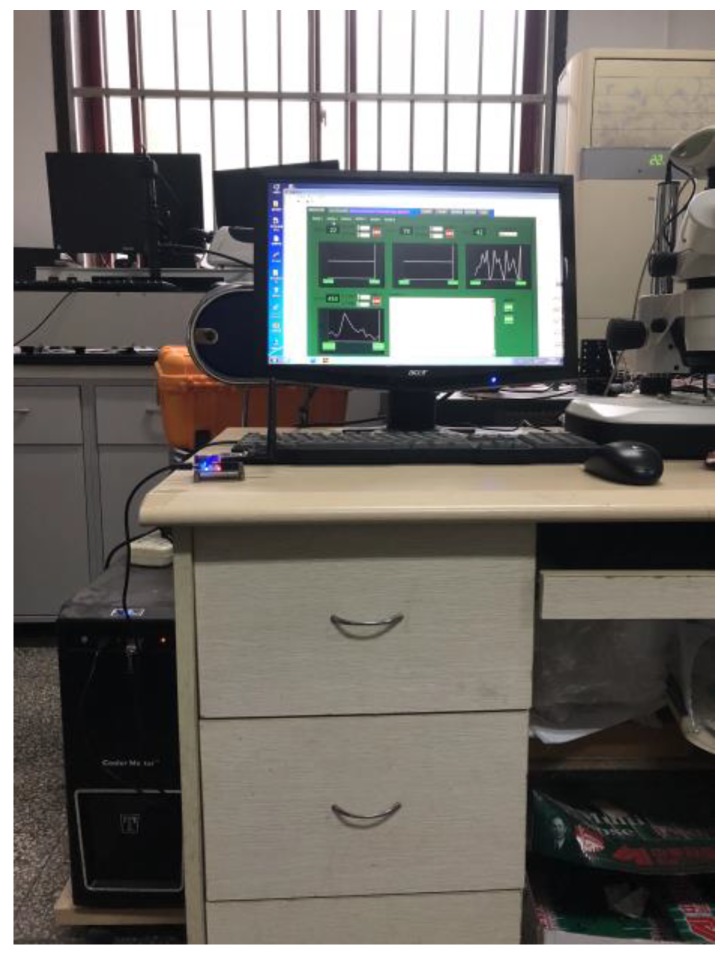
The local monitoring platform.

The ancient building that we monitored is located in Xi’an, Shaanxi Province, China, which is the provincial unit of cultural relics protection. As an ancient building with a brick (stone) structure, it is affected by environmental parameters, such as the temperature, relative humidity, sulfur dioxide, vibration, and so on. The monitored building is shown in Figure 9, and this system monitors six rooms locally, and two rooms for the remote monitoring of the environment. Furthermore, this system also monitors the parameters of the outdoor environment. As shown in Figure 10 and Figure 11, this system has two monitoring platforms, namely: a remote monitoring platform named the sever, and a local monitoring platform named the client. The server can only receive the remote data, but the client can accept the remote data and local data.

#### 3.2.2. Login System

**Figure 12:**
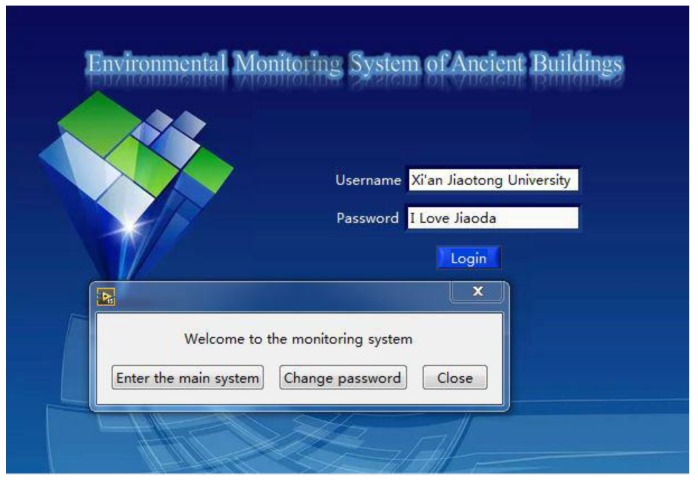
The login system.

This paper describes the design of the login system. As shown in Figure 12, the login system includes the following three parts: “Enter the main system”, “Change password”, and “Close”. The username set in this system is “Xi’an Jiaotong University”, and the password is “I Love Jiaoda”.

#### 3.2.3. Main System

**Figure 13:**
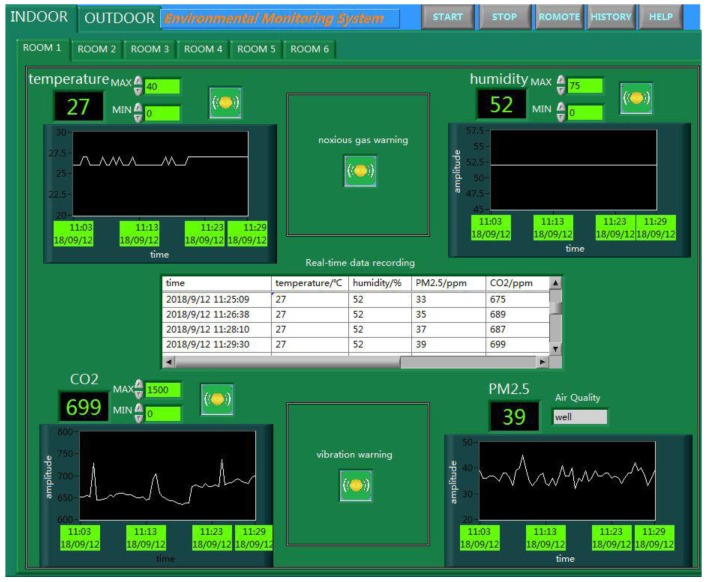
The client system.

**Figure 14:**
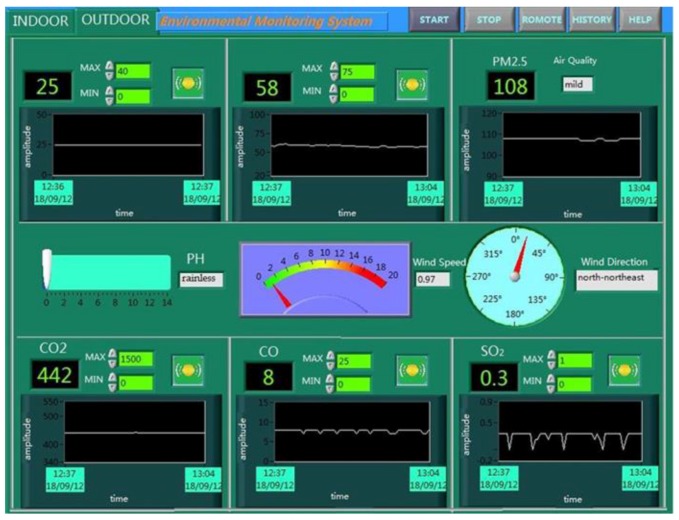
The monitoring of outdoor environment.

The main system is the core of the entire monitoring platform, and is also divided into the server system and the client system. As shown in Figure 13, the client system can receive and process the monitoring data of the entire system. For the environmental parameters monitored indoors, such as the temperature, relative humidity, PM2.5, and carbon dioxide, we can realize the real-time display, storage, and over-limit warning. But for the vibration and liquefied gas, we can only achieve the function of over-limit warning because of the limitations of the sensor itself. Furthermore, the monitoring of the outdoor environment is more complicated than for indoors; its specific monitoring interface is shown in Figure 14.

**Figure 15:**
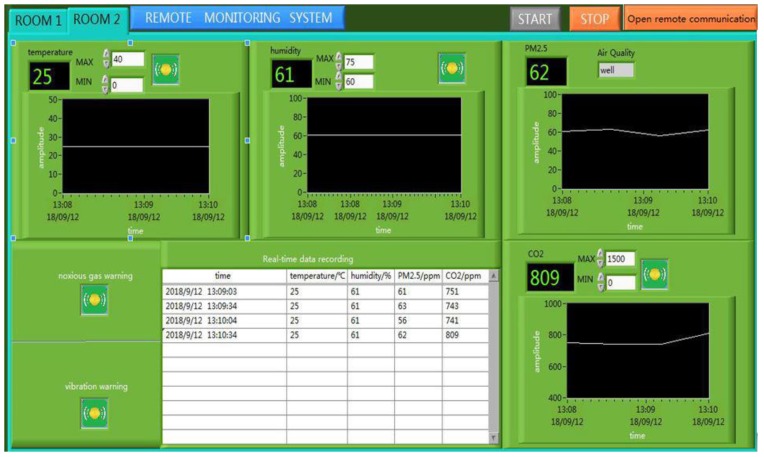
The server system.

**Figure 16:**
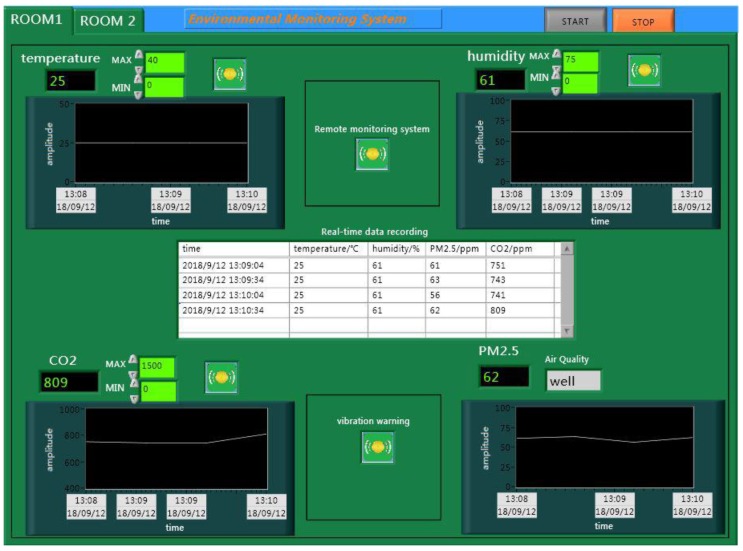
The remote monitoring interface.

As shown in Figure 15, the server system is similar to the client system. When you click on the “Open remote communication” of the server system, and the “REMOTE” of the client system, the client system will accept the data of the remote monitoring. The remote monitoring interface of the client system is shown in Figure 16.

**Figure 17:**
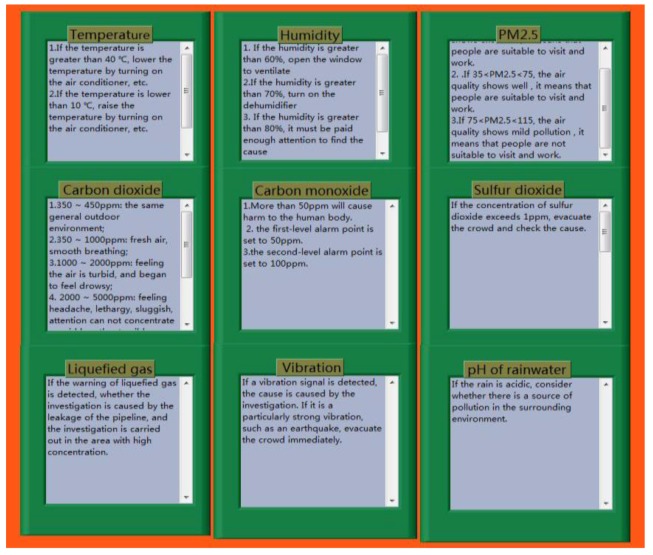
The “HELP” menu.

**Figure 18:**
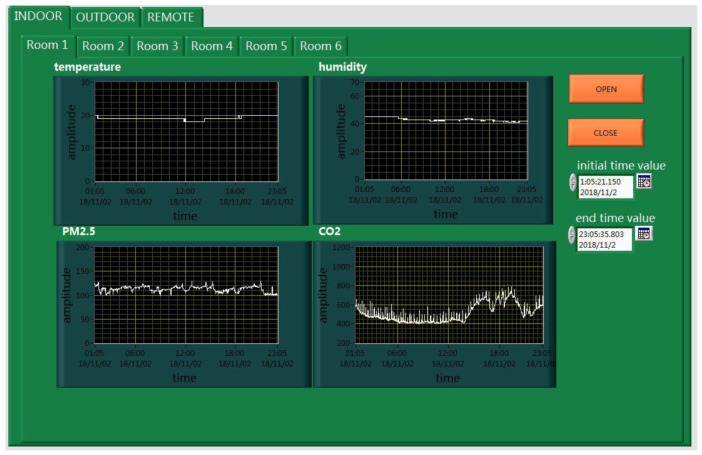
The “HISTORY” menu.

In the process of environmental monitoring, we have developed corresponding plans in advance for any problems that may be encountered. As shown in Figure 17, people can view various notes on the “HELP” menu, and can directly view the curve changes of the monitoring data by setting the start time and end time, as shown in Figure 18.

### 3.3. Data Analysis

**Figure 19:**
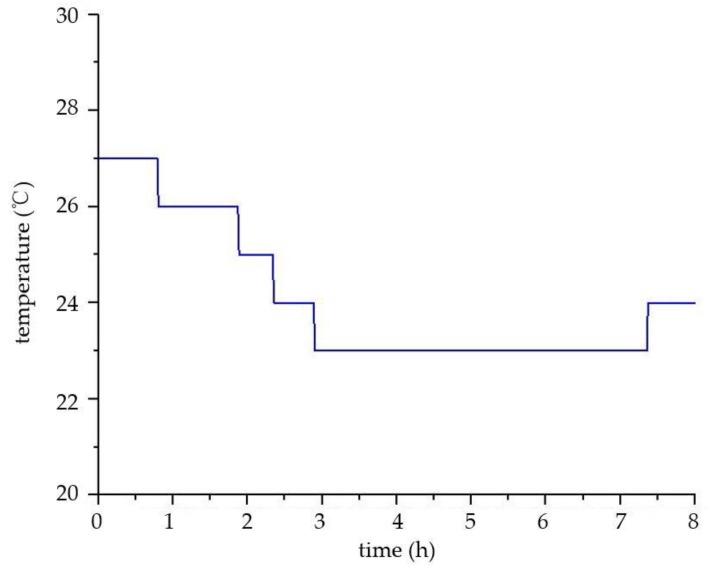
The curve of temperature.

**Figure 20:**
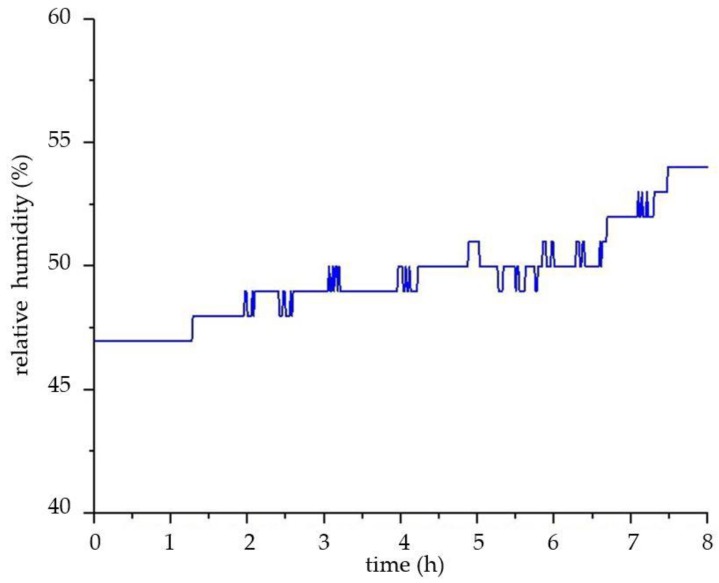
The curve of relative humidity.

**Figure 21:**
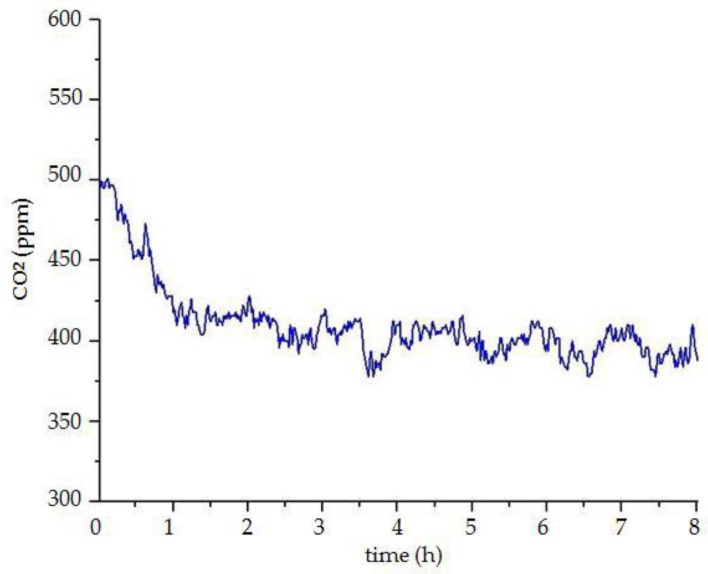
The curve of carbon dioxide concentration.

**Figure 22:**
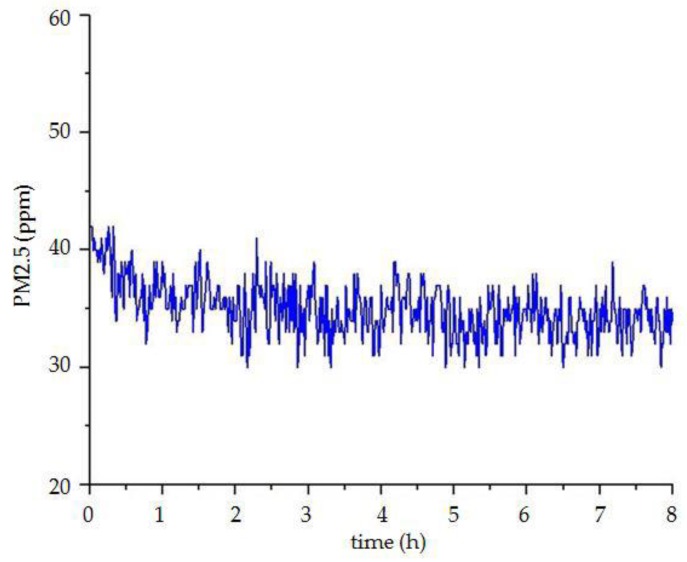
The curve of PM2.5 concentration.

The focus of the environmental monitoring of ancient buildings is to provide a scientific basis for the protection of ancient buildings, by collating and analyzing the environmental data. This paper simulates the environmental monitoring of ancient buildings and conducts the data analysis simply. The environmental data selected is from 12 p.m. to 8 a.m. on a certain day in Xi’an City, Shaanxi Province, China. As shown in Figure 19 and Figure 20, the temperature is constantly decreasing and the relative humidity increases slowly. These are consistent with the increase of relative humidity and the decline of temperature in the evening in Xi’an. As shown in Figure 21, the concentration of carbon dioxide decreases at the beginning, and then begins to level off. The concentration change of PM2.5 is also similar to carbon dioxide, as shown in Figure 22. The reasons for their changes are related to the lack of personnel activities at night.

**Figure 23:**
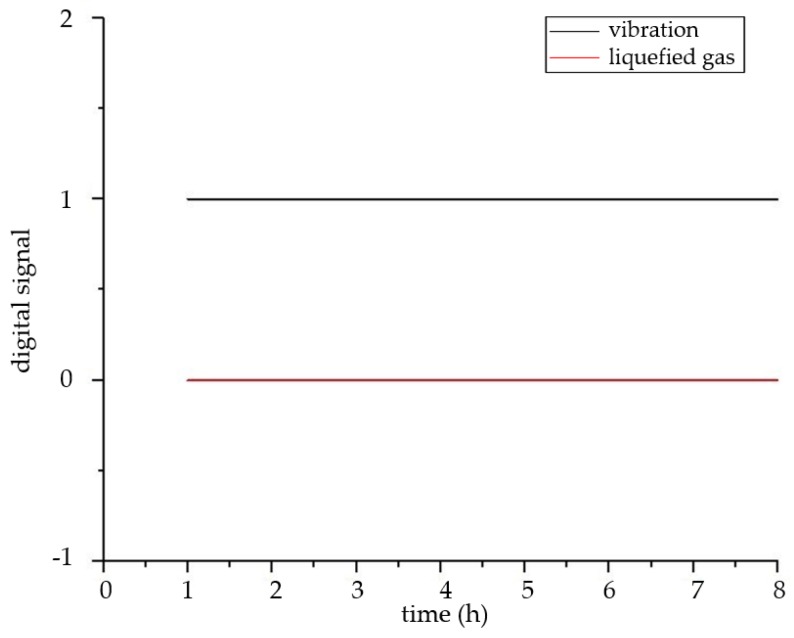
The curve of vibration and toxic gas.

**Figure 24:**
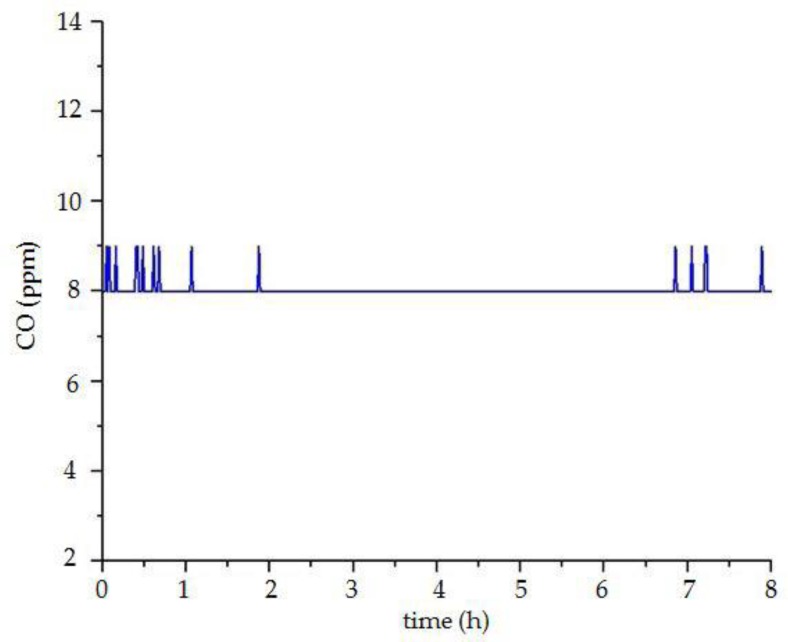
The curve of carbon monoxide concentration.

**Figure 25:**
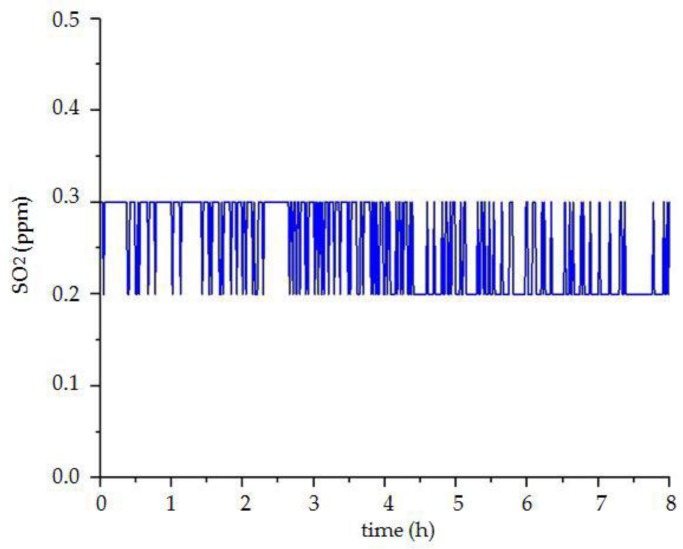
The curve of sulfur dioxide concentration.

The system uses the mode of over-limit warning for monitoring vibration and toxic gas. The vibration sensor and liquefied gas sensor use the transmission of the switching signal. When the liquefied gas is detected, the sensor outputs the number “1”. If there is no toxic gas, the sensor outputs the number “0”. The setting of the vibration sensor is opposite to the liquefied gas sensor. When there is vibration, the number “0” is output, and when there is no vibration, the number “1” is output. It can be seen from the experimental results in Figure 23 that the liquefied gas sensor outputs the signal of ”0”, and the vibration sensor outputs the signal of “1”. These results show that there is no liquefied gas and vibration in the room, which is consistent with the actual situation. Moreover, the concentration changes of carbon monoxide and sulfur dioxide are shown in Figure 24 and Figure 25, and their concentrations are normal and the changes are very stable.

**Figure 26:**
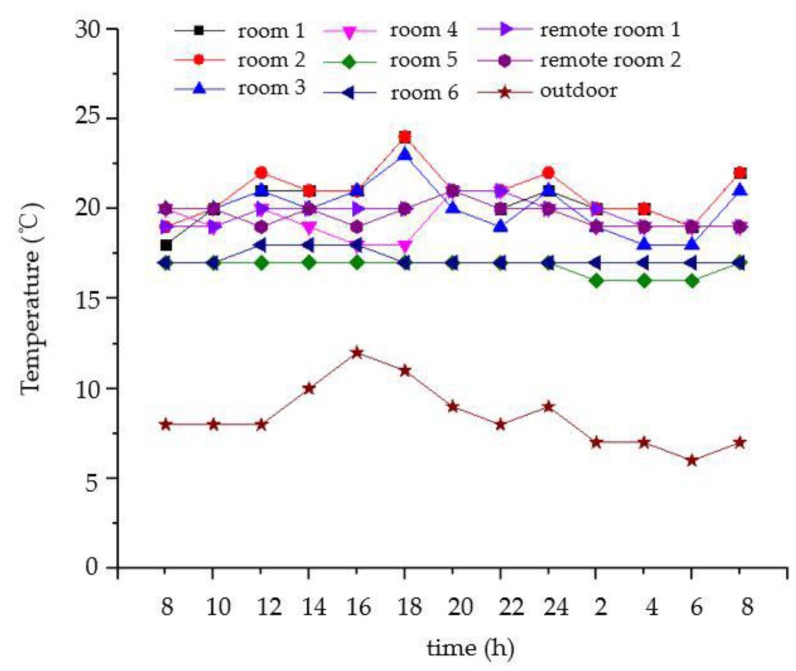
The curve of the temperature in different rooms on the same day.

**Figure 27:**
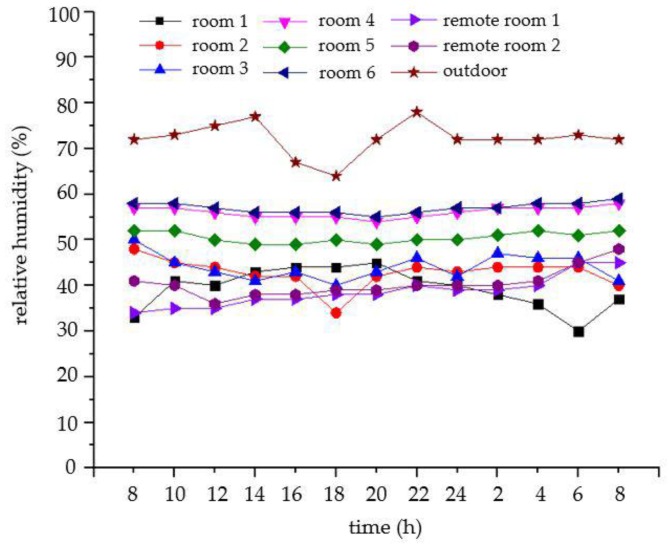
The curve of the relative humidity in different rooms on the same day.

**Figure 28:**
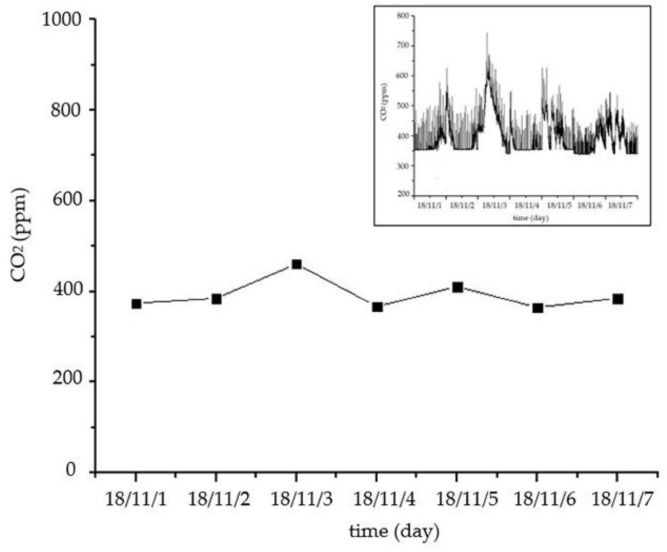
The curve of carbon dioxide concentration in a week.

**Figure 29:**
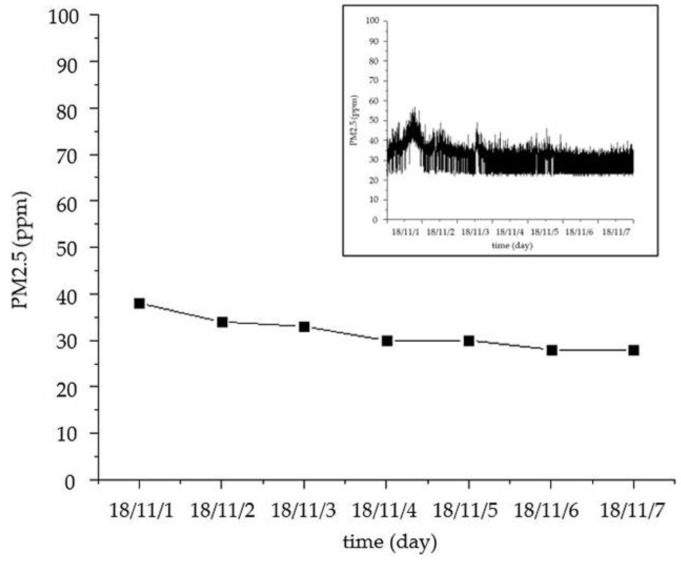
The curve of PM2.5 concentration in a week.

As shown in Figure 26 and Figure 27, the temperature and relative humidity of the different rooms are slightly different on the same day, and the changes are smooth. For the outdoor temperature and relative humidity, their changes are also normal. The curves of the carbon dioxide and PM2.5 concentrations in a week are shown in Figure 28 and Figure 29, respectively. We average the monitored data every day. The concentrations of carbon dioxide and PM2.5 were around 400 ppm and 35 ppm, respectively, and their changes were very stable. Therefore, the environmental data in the ancient building using this monitoring system is stable. The long-term monitoring also proves the reliability and stability of the monitoring system.

## 4. Discussion

This paper describes a wireless monitoring system for ancient buildings. For the requirements of the multi-node and multi-sensor, this monitoring system designs the new indoor node package box and outdoor package device. Furthermore, this system monitors eleven types of environmental parameters, and designs a powerful online monitoring platform. At present, most environmental monitoring systems have the following problems: a single monitoring parameter, single function of the monitoring software, no packaging design, and so on. The comparison of this monitoring system with other monitoring systems is shown in Table 3.

In addition to considering the impact of the environmental parameters on ancient buildings, the impact on people is also considered. For example, this system monitors the concentration of PM2.5, which has a significant impact on people’s health. We divided the value of the PM2.5 concentration into five grades, corresponding to the different grades of air quality, as shown in Table 4. The monitoring platform can display both the concentration value of PM2.5 and the corresponding air quality level. Moreover, this monitoring system can also realize the function of fire warning by monitoring liquefied gas, and so on. Unfortunately, because of the leaking of the pipeline gas, a very serious fire occurred in 2013 in the ancient building monitored in this paper. If the gas leakage could have been detected in time, the fire may not have happened.

Because the system needs to monitor a lot of environmental parameters, it has higher requirements on the reliability and stability of the network, especially in the long-term management of this system. Because of ZigBee’s feature of a self-organizing network, the terminal node can enter the network through the router or coordinator according to the strength of the signal. Therefore, we can use a large number of routers to extend the transmission distance and improve the reliability of the network. If a router is damaged, the terminal node can also transmit the data through other routers, which greatly increases the reliability and stability of the system. In addition, some of the sensors used in this system, such as the vibration sensor and liquefied gas sensor, have low precision because of the limitations of the sensors themselves, considering the low cost. This has an impact on long-term research. This system can be directly replaced with the related sensors of higher-precision, without any impact on the entire system. In addition, ZigBee technology is also widely used in the field of energy saving projects. Many experts, such as Cagnetti [42], Liu [43], and so on, have also done a lot of work on this topic. It is also very interesting to note the function of energy dissipation monitoring in this system.

The monitoring system of this paper is applied in an ancient building with a brick (stone) structure. However, as far as this monitoring system is concerned, it can also be applied to other ancient buildings. In the future, we will apply this monitoring system to more ancient buildings, in order to explore more possibilities.

## 5. Conclusions

In order to achieve the goal of providing data support for the preventive protection programs of ancient buildings, a complete system with a multi-sensor and multi-node, for the environmental monitoring of ancient buildings is proposed, from the construction of hardware to designs of the monitoring platform and packaging. This system monitors eleven types of environmental parameters and uses ten types of sensors. For the needs of ancient buildings, the new indoor node package box and outdoor package device were designed and manufactured. For the indoor node package box, the layered structure design can isolate the node module from the sensor module, which can not only protect the node module from dust and water, but can also facilitate replacement in the case of damage to the sensor module. For the design of the outdoor package device, it also has functions of being waterproof and dustproof, especially the realization of rainwater collection and discharge. We also designed an online monitoring platform with functions of real-time display, storage, over-limit warning, and so on. People can view the related information and all of the monitored data through the “HELP” and “HISTORY” menu, respectively. It is has also been proven that the monitoring system is stable and reliable, by monitoring the indoor and outdoor environmental information of actual ancient buildings.

## Figures and Tables

**Table 1:** The types of sensors and technical parameters used in the monitoring system.

Name of Sensor	Scope of Monitoring	Accuracy of Measurement	Type of Sensor
Relative humidity and temperature sensor	0–50 °C	±2 °C	DHT11
	20–95%	±5%	
PM2.5 sensor	0–8000 pcs	±1 um	GP2Y1014AU0F
Carbon dioxide sensor	0–2000 ppm	±50 ppm	MH-Z19B
Carbon monoxide sensor	0–500 ppm	±1 ppm	AJD-4CO
Sulfur dioxide sensor	0–20 ppm	±0.1 ppm	AJD-4SO2
liquefied gas sensor	0/1		MQ-2
Vibration sensor	0/1		SW-1801P
Wind speed sensor	0–70 m/s	±0.3 m/s	YGC-FS
Wind direction sensor	0–359°	±3°	YGC-FX
pH sensor	0–14	±0.01	E-201-C

**Table 2:** The connection between the signal output of the sensor and the signal input of the terminal device.

**Connection between Indoor Terminal Device and Sensor**	
**Name of Sensor**	**Indoor Terminal Device**
Relative humidity and Temperature sensor	P07, GND, 5 V
PM2.5 sensor	P06, P10, GND, 5 V
Carbon dioxide sensor	P04, GND, 5 V
Liquefied gas sensor	P05, GND, 5 V
Vibration sensor	P12, GND, 3.3 V
**Connection between Outdoor Terminal Device 1 and Sensor**	
**Name of Sensor**	**Outdoor Terminal Device 1**
Relative humidity and temperature sensor	P07, GND, 5 V
PM2.5 sensor	P06, P10, GND, 5 V
Carbon dioxide sensor	P05, GND, 5 V
Carbon monoxide sensor	P04, GND, 3.3 V
Sulfur dioxide sensor	P00, GND, 3.3 V
**Connection between Outdoor Terminal Device 2 and Sensor**	
**Name of Sensor**	**Outdoor Terminal Device 2**
Wind speed sensor	P04, GND, 5 V
Wind direction sensor	P06, GND, 5 V
pH sensor	P05, GND, 5 V

**Table 3:** The comparison of this monitoring system with other monitoring systems.

Monitoring System	Monitoring Parameter	Monitoring Software	Packaging Design
Wu et al. [20]	Smoke	Display and warning	No packaging design
Pérez-Garrido et al. [23]	Toxic gases	No design software	Simple
Zhang et al. [34]	Temperature, relative humidity, and carbon dioxide	Simple	Simple
Agbota et al. [37]	Alternative materials	Simple	Simple
Zhao et al. [38]	Electrical fire	No design software	Simple
This paper	Eleven parameters	Complete function	Two kinds of packaging

**Table 4:** The grades of air quality.

Scope of PM2.5 Concentration	Air Quality
0 < PM2.5 ≤ 35	Excellent
35 < PM2.5 ≤ 75	Well
75 < PM2.5 ≤ 115	Mild pollution
115 < PM2.5 ≤ 150	Moderate pollution
150 < PM2.5 ≤ 250	Severe contamination
